# *catena*-Poly[[pyridinecopper(II)]-μ-*N*-[(2-oxido-1-naphth­yl)methyl­ene]glycinato]

**DOI:** 10.1107/S1600536809037520

**Published:** 2009-09-26

**Authors:** Ling-Wei Xue, Xing-Wu Li, Gan-Qing Zhao, Qin-Long Peng

**Affiliations:** a School of Chemistry and Chemical Engineering, Pingdingshan University, Pingdingshan 467000, People’s Republic of China

## Abstract

In the title compound, [Cu(C_13_H_9_NO_3_)(C_5_H_5_N)], the Cu^II^ atom is coordinated in a distorted square-pyramidal geometry, with two N and two O atoms in the basal positions and one O atom in the apical position. The apical Cu—O bond [2.3520 (16) Å] is much longer than the basal Cu—O and Cu—N bonds [1.9139 (14)–2.0136 (17) Å]. The carboxyl­ate group bridges Cu^II^ atoms, forming a zigzag chain along the *a* axis.

## Related literature

For related structures, see: Basu Baul *et al.* (2007 ▶); Parekh *et al.* (2006 ▶); Usman *et al.* (2003 ▶); Vigato & Tamburini (2004 ▶); Casella & Gullotti (1983 ▶).
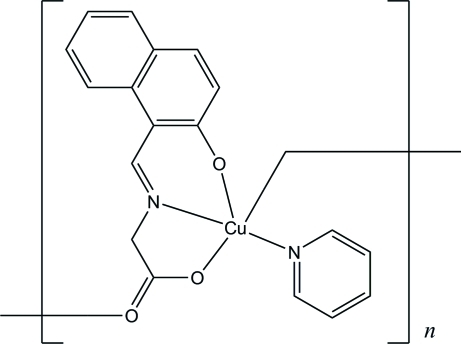


## Experimental

### 

#### Crystal data

[Cu(C_13_H_9_NO_3_)(C_5_H_5_N)]
*M* *_r_* = 369.85
Monoclinic, 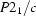

*a* = 14.508 (4) Å
*b* = 11.747 (3) Å
*c* = 9.407 (3) Å
β = 101.805 (3)°
*V* = 1569.5 (8) Å^3^
*Z* = 4
Mo *K*α radiation
μ = 1.41 mm^−1^
*T* = 296 K
0.30 × 0.30 × 0.25 mm

#### Data collection

Bruker APEXII CCD diffractometer
Absorption correction: multi-scan (**SADABS**; Sheldrick, 1996 ▶) *T*_min_ = 0.662, *T*_max_ = 0.703
7938 measured reflections
2770 independent reflections
2460 reflections with *I* > 2σ(*I*)
*R*_int_ = 0.018

#### Refinement

*R*[*F* ^2^ > 2σ(*F* ^2^)] = 0.026
*wR*(*F* ^2^) = 0.069
*S* = 1.04
2770 reflections
218 parameters
H-atom parameters constrained
Δρ_max_ = 0.26 e Å^−3^
Δρ_min_ = −0.23 e Å^−3^

### 

Data collection: *APEX2* (Bruker, 2005 ▶); cell refinement: *SAINT* (Bruker, 2005 ▶); data reduction: *SAINT*; program(s) used to solve structure: *SHELXS97* (Sheldrick, 2008 ▶); program(s) used to refine structure: *SHELXL97* (Sheldrick, 2008 ▶); molecular graphics: *SHELXTL* (Sheldrick, 2008 ▶); software used to prepare material for publication: *SHELXTL*.

## Supplementary Material

Crystal structure: contains datablocks global, I. DOI: 10.1107/S1600536809037520/is2450sup1.cif

Structure factors: contains datablocks I. DOI: 10.1107/S1600536809037520/is2450Isup2.hkl

Additional supplementary materials:  crystallographic information; 3D view; checkCIF report

## Figures and Tables

**Table 1:** Selected bond lengths (Å)

Cu1—O1	1.9139 (14)
Cu1—N1	1.9296 (17)
Cu1—O2	1.9702 (14)
Cu1—N2	2.0136 (17)
Cu1—O3^i^	2.3520 (16)

Symmetry code: (i) 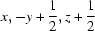.

**Fig. 1.**
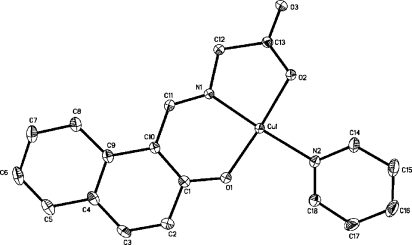
The structure of the title compound, showing 30% probability displacement ellipsoids and the atom-numbering scheme.

**Fig. 2.**
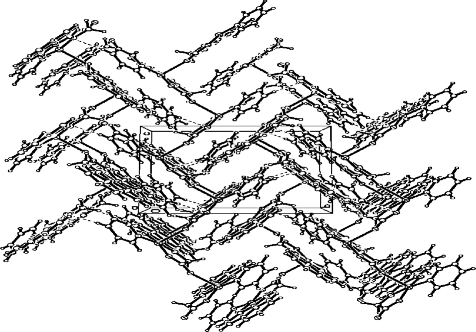
A view of the crystal packing along the c axis.

## supplementary crystallographic information

### Comment

In the past decades, significant progress has been achieved in understanding the chemistry of transition metal complexes with Schiff base ligands composed of salicylaldehyde, 2-formylpyridine or their analogues, and α-amino acids (Vigato & Tamburini, 2004; Casella & Gullotti, 1983). A few structural studies have been performed on Schiff base complexes derived from 2-hydroxyacetophenone and animo acids (Usman *et al.*, 2003; Basu Baul *et al.*, 2007; Parekh *et al.*, 2006). We report here the crystal structure of the title Cu^II^ complex, (I).

The structure consists of a square pyramidal Cu^II^ complex (Fig. 1 and Table 1). The four basal positions are occupied by three donor atoms from the tridentate Schiff base ligand, which furnishes an ONO donor set, with the fourth position occupied by one N atom from the pyridine ligand. The fifth position is occupied by one O atom from the adjacent tridentate Schiff base ligand.

The crystal structure is stabilized by the long-distance coordination of Cu1 and O3 (Fig. 2 and Table 2). The distance of Cu1—O3 bonds is 2.3520 (16) Å, and the distance of the two Cu(II) atoms is 6.013 Å.

### Experimental

The title compound was synthesized as described in the literature. To glycine (1.00 mmol) and potassium hydroxide (1.00 mmol) in 10 ml of methanol and 5 ml of water was added 2-hydroxy-1-naphthaldehyde (1.00 mmol in 10 ml of methanol) dropwise. The yellow solution was stirred for 2.0 h at 333 K. The resultant mixture was added dropwise to Cu(II) nitrate hexahydrate (1.00 mmol) and pyridine (1.00 mmol) in an aqueous methanolic solution (20 ml, 1:1 *v*/*v*), and heated with stirring for 2.0 h at 333 K. The brown solution was filtered and left for several days, brown crystals had formed that were filtered off, washed with water, and dried under vacuum.

### Refinement

All H atoms were positioned geometrically and refined as riding atoms, with C—H = 0.93 or 0.97 Å, and with *U*_iso_(H) = 1.2*U*_eq_(C).

### Crystal data

**Table:**

[Cu(C_13_H_9_NO_3_)(C_5_H_5_N)]	*F*(000) = 756
*M**_r_* = 369.85	*D*_x_ = 1.565 Mg m^−^^3^
Monoclinic, *P*2_1_/*c*	Mo *K*α radiation, λ = 0.71073 Å
Hall symbol: -P 2ybc	Cell parameters from 4385 reflections
*a* = 14.508 (4) Å	θ = 2.3–27.5°
*b* = 11.747 (3) Å	µ = 1.41 mm^−^^1^
*c* = 9.407 (3) Å	*T* = 296 K
β = 101.805 (3)°	Block, brown
*V* = 1569.5 (8) Å^3^	0.30 × 0.30 × 0.25 mm
*Z* = 4	

### Data collection

**Table:**

Bruker APEXII CCD diffractometer	2770 independent reflections
Radiation source: fine-focus sealed tube	2460 reflections with *I* > 2σ(*I*)
graphite	*R*_int_ = 0.018
φ and ω scans	θ_max_ = 25.0°, θ_min_ = 2.3°
Absorption correction: multi-scan (*SADABS*; Sheldrick, 1996)	*h* = −15→17
*T*_min_ = 0.662, *T*_max_ = 0.703	*k* = −13→13
7938 measured reflections	*l* = −10→11

### Refinement

**Table:**

Refinement on *F*^2^	Secondary atom site location: difference Fourier map
Least-squares matrix: full	Hydrogen site location: inferred from neighbouring sites
*R*[*F*^2^ > 2σ(*F*^2^)] = 0.026	H-atom parameters constrained
*wR*(*F*^2^) = 0.069	*w* = 1/[σ^2^(*F*_o_^2^) + (0.0336*P*)^2^ + 0.6894*P*] where *P* = (*F*_o_^2^ + 2*F*_c_^2^)/3
*S* = 1.04	(Δ/σ)_max_ = 0.001
2770 reflections	Δρ_max_ = 0.26 e Å^−^^3^
218 parameters	Δρ_min_ = −0.23 e Å^−^^3^
0 restraints	Extinction correction: *SHELXL97* (Sheldrick, 2008), Fc^*^=kFc[1+0.001xFc^2^λ^3^/sin(2θ)]^-1/4^
Primary atom site location: structure-invariant direct methods	Extinction coefficient: 0.0072 (6)

### Special details

**Table:**

Geometry. All e.s.d.'s (except the e.s.d. in the dihedral angle between two l.s. planes) are estimated using the full covariance matrix. The cell e.s.d.'s are taken into account individually in the estimation of e.s.d.'s in distances, angles and torsion angles; correlations between e.s.d.'s in cell parameters are only used when they are defined by crystal symmetry. An approximate (isotropic) treatment of cell e.s.d.'s is used for estimating e.s.d.'s involving l.s. planes.
Refinement. Refinement of *F*^2^ against ALL reflections. The weighted *R*-factor *wR* and goodness of fit *S* are based on *F*^2^, conventional *R*-factors *R* are based on *F*, with *F* set to zero for negative *F*^2^. The threshold expression of *F*^2^ > σ(*F*^2^) is used only for calculating *R*-factors(gt) *etc*. and is not relevant to the choice of reflections for refinement. *R*-factors based on *F*^2^ are statistically about twice as large as those based on *F*, and *R*- factors based on ALL data will be even larger.

### Fractional atomic coordinates and isotropic or equivalent isotropic displacement parameters (Å^2^)

**Table:**

	*x*	*y*	*z*	*U*_iso_*/*U*_eq_	
Cu1	0.754799 (16)	0.40947 (2)	0.14007 (3)	0.03547 (11)	
C1	0.58094 (14)	0.47447 (17)	0.2269 (2)	0.0363 (5)	
C2	0.53738 (17)	0.5434 (2)	0.3195 (3)	0.0499 (6)	
H2	0.5734	0.5967	0.3797	0.060*	
C3	0.44485 (18)	0.5327 (2)	0.3216 (3)	0.0555 (6)	
H3	0.4194	0.5774	0.3855	0.067*	
C4	0.38571 (16)	0.4559 (2)	0.2299 (3)	0.0468 (6)	
C5	0.28967 (18)	0.4444 (3)	0.2361 (3)	0.0638 (7)	
H5	0.2657	0.4864	0.3041	0.077*	
C6	0.23200 (19)	0.3736 (3)	0.1457 (4)	0.0716 (8)	
H6	0.1693	0.3659	0.1525	0.086*	
C7	0.26763 (17)	0.3122 (2)	0.0417 (3)	0.0645 (7)	
H7	0.2279	0.2647	−0.0224	0.077*	
C8	0.36069 (15)	0.3211 (2)	0.0331 (3)	0.0494 (6)	
H8	0.3827	0.2801	−0.0378	0.059*	
C9	0.42367 (15)	0.39105 (17)	0.1292 (2)	0.0389 (5)	
C10	0.52409 (14)	0.39925 (16)	0.1297 (2)	0.0335 (4)	
C11	0.56303 (14)	0.33185 (17)	0.0311 (2)	0.0354 (5)	
H11	0.5214	0.2884	−0.0357	0.042*	
C12	0.68024 (15)	0.25177 (19)	−0.0810 (2)	0.0436 (5)	
H12A	0.6376	0.2612	−0.1742	0.052*	
H12B	0.6775	0.1729	−0.0515	0.052*	
C13	0.77987 (14)	0.28074 (17)	−0.0955 (2)	0.0358 (5)	
C14	0.93488 (18)	0.5307 (3)	0.1688 (3)	0.0643 (7)	
H14	0.9353	0.4959	0.0802	0.077*	
C15	1.0076 (2)	0.6014 (3)	0.2262 (4)	0.0809 (10)	
H15	1.0560	0.6146	0.1768	0.097*	
C16	1.00878 (19)	0.6523 (3)	0.3557 (4)	0.0771 (9)	
H16	1.0572	0.7017	0.3957	0.093*	
C17	0.9372 (2)	0.6294 (3)	0.4269 (4)	0.0774 (9)	
H17	0.9371	0.6613	0.5173	0.093*	
C18	0.86512 (17)	0.5579 (2)	0.3615 (3)	0.0601 (7)	
H18	0.8161	0.5436	0.4090	0.072*	
N1	0.65107 (11)	0.32565 (14)	0.02613 (18)	0.0353 (4)	
N2	0.86312 (12)	0.50919 (16)	0.2340 (2)	0.0424 (4)	
O1	0.67150 (10)	0.48556 (12)	0.24244 (16)	0.0414 (3)	
O2	0.82497 (9)	0.34878 (13)	−0.00179 (15)	0.0400 (3)	
O3	0.81199 (11)	0.23568 (14)	−0.19287 (17)	0.0514 (4)	

### Atomic displacement parameters (Å^2^)

**Table:**

	*U*^11^	*U*^22^	*U*^33^	*U*^12^	*U*^13^	*U*^23^
Cu1	0.03099 (16)	0.03984 (17)	0.03662 (17)	−0.00672 (10)	0.00934 (11)	−0.00578 (11)
C1	0.0389 (12)	0.0339 (11)	0.0372 (11)	0.0025 (9)	0.0102 (9)	0.0061 (9)
C2	0.0500 (14)	0.0490 (14)	0.0523 (14)	0.0021 (11)	0.0147 (11)	−0.0094 (11)
C3	0.0544 (15)	0.0599 (15)	0.0579 (16)	0.0146 (12)	0.0247 (12)	−0.0053 (13)
C4	0.0391 (12)	0.0486 (13)	0.0557 (14)	0.0110 (11)	0.0164 (11)	0.0125 (11)
C5	0.0460 (15)	0.0705 (17)	0.081 (2)	0.0161 (14)	0.0276 (14)	0.0127 (15)
C6	0.0327 (13)	0.0795 (19)	0.106 (2)	0.0041 (14)	0.0221 (15)	0.0161 (18)
C7	0.0344 (13)	0.0645 (16)	0.092 (2)	−0.0042 (12)	0.0073 (13)	0.0084 (15)
C8	0.0360 (12)	0.0474 (13)	0.0645 (16)	0.0012 (10)	0.0095 (11)	0.0067 (12)
C9	0.0339 (11)	0.0350 (11)	0.0482 (13)	0.0049 (9)	0.0094 (9)	0.0136 (9)
C10	0.0315 (11)	0.0326 (10)	0.0367 (11)	0.0026 (8)	0.0080 (8)	0.0072 (8)
C11	0.0324 (11)	0.0352 (11)	0.0375 (11)	−0.0038 (9)	0.0045 (8)	0.0014 (9)
C12	0.0375 (12)	0.0467 (13)	0.0483 (13)	−0.0069 (10)	0.0130 (9)	−0.0136 (10)
C13	0.0360 (11)	0.0367 (11)	0.0354 (11)	−0.0006 (9)	0.0089 (9)	0.0009 (9)
C14	0.0480 (15)	0.083 (2)	0.0647 (17)	−0.0268 (14)	0.0184 (13)	−0.0101 (15)
C15	0.0530 (17)	0.098 (2)	0.093 (2)	−0.0358 (16)	0.0169 (16)	−0.0089 (19)
C16	0.0423 (16)	0.0655 (19)	0.114 (3)	−0.0164 (14)	−0.0072 (16)	−0.0147 (18)
C17	0.0533 (17)	0.084 (2)	0.087 (2)	−0.0036 (15)	−0.0049 (15)	−0.0427 (18)
C18	0.0399 (14)	0.0733 (17)	0.0659 (17)	−0.0066 (12)	0.0079 (12)	−0.0227 (14)
N1	0.0324 (9)	0.0383 (9)	0.0365 (9)	−0.0034 (7)	0.0102 (7)	−0.0055 (7)
N2	0.0355 (10)	0.0449 (10)	0.0458 (11)	−0.0082 (8)	0.0059 (8)	−0.0046 (8)
O1	0.0363 (8)	0.0436 (8)	0.0452 (8)	−0.0046 (6)	0.0106 (6)	−0.0091 (7)
O2	0.0334 (8)	0.0491 (9)	0.0390 (8)	−0.0071 (7)	0.0107 (6)	−0.0067 (7)
O3	0.0468 (9)	0.0618 (10)	0.0512 (9)	−0.0097 (8)	0.0233 (7)	−0.0191 (8)

### Geometric parameters (Å, °)

**Table:**

Cu1—O1	1.9139 (14)	C9—C10	1.459 (3)
Cu1—N1	1.9296 (17)	C10—C11	1.422 (3)
Cu1—O2	1.9702 (14)	C11—N1	1.290 (3)
Cu1—N2	2.0136 (17)	C11—H11	0.9300
Cu1—O3^i^	2.3520 (16)	C12—N1	1.457 (3)
C1—O1	1.298 (2)	C12—C13	1.518 (3)
C1—C10	1.410 (3)	C12—H12A	0.9700
C1—C2	1.428 (3)	C12—H12B	0.9700
C2—C3	1.352 (3)	C13—O3	1.229 (2)
C2—H2	0.9300	C13—O2	1.268 (2)
C3—C4	1.411 (4)	C14—N2	1.336 (3)
C3—H3	0.9300	C14—C15	1.365 (4)
C4—C9	1.412 (3)	C14—H14	0.9300
C4—C5	1.413 (3)	C15—C16	1.354 (5)
C5—C6	1.350 (4)	C15—H15	0.9300
C5—H5	0.9300	C16—C17	1.373 (4)
C6—C7	1.397 (4)	C16—H16	0.9300
C6—H6	0.9300	C17—C18	1.383 (4)
C7—C8	1.373 (3)	C17—H17	0.9300
C7—H7	0.9300	C18—N2	1.324 (3)
C8—C9	1.411 (3)	C18—H18	0.9300
C8—H8	0.9300	O3—Cu1^ii^	2.3520 (16)
			
O1—Cu1—N1	90.96 (7)	C1—C10—C9	119.63 (19)
O1—Cu1—O2	167.63 (6)	C11—C10—C9	119.40 (19)
N1—Cu1—O2	83.77 (6)	N1—C11—C10	125.71 (19)
O1—Cu1—N2	91.38 (7)	N1—C11—H11	117.1
N1—Cu1—N2	172.14 (7)	C10—C11—H11	117.1
O2—Cu1—N2	92.44 (7)	N1—C12—C13	110.20 (17)
O1—Cu1—O3^i^	100.09 (6)	N1—C12—H12A	109.6
N1—Cu1—O3^i^	97.45 (7)	C13—C12—H12A	109.6
O2—Cu1—O3^i^	91.71 (6)	N1—C12—H12B	109.6
N2—Cu1—O3^i^	89.52 (7)	C13—C12—H12B	109.6
O1—C1—C10	125.43 (19)	H12A—C12—H12B	108.1
O1—C1—C2	116.02 (19)	O3—C13—O2	124.69 (19)
C10—C1—C2	118.5 (2)	O3—C13—C12	118.91 (19)
C3—C2—C1	121.4 (2)	O2—C13—C12	116.38 (17)
C3—C2—H2	119.3	N2—C14—C15	123.1 (3)
C1—C2—H2	119.3	N2—C14—H14	118.5
C2—C3—C4	122.1 (2)	C15—C14—H14	118.5
C2—C3—H3	118.9	C16—C15—C14	119.4 (3)
C4—C3—H3	118.9	C16—C15—H15	120.3
C3—C4—C9	118.9 (2)	C14—C15—H15	120.3
C3—C4—C5	121.2 (2)	C15—C16—C17	118.8 (3)
C9—C4—C5	119.9 (2)	C15—C16—H16	120.6
C6—C5—C4	121.5 (3)	C17—C16—H16	120.6
C6—C5—H5	119.3	C16—C17—C18	118.7 (3)
C4—C5—H5	119.3	C16—C17—H17	120.6
C5—C6—C7	119.3 (2)	C18—C17—H17	120.6
C5—C6—H6	120.4	N2—C18—C17	122.7 (3)
C7—C6—H6	120.4	N2—C18—H18	118.7
C8—C7—C6	120.8 (3)	C17—C18—H18	118.7
C8—C7—H7	119.6	C11—N1—C12	119.25 (17)
C6—C7—H7	119.6	C11—N1—Cu1	128.09 (14)
C7—C8—C9	121.5 (2)	C12—N1—Cu1	112.60 (13)
C7—C8—H8	119.3	C18—N2—C14	117.3 (2)
C9—C8—H8	119.3	C18—N2—Cu1	121.30 (16)
C8—C9—C4	117.0 (2)	C14—N2—Cu1	121.35 (17)
C8—C9—C10	123.8 (2)	C1—O1—Cu1	128.69 (13)
C4—C9—C10	119.2 (2)	C13—O2—Cu1	115.74 (12)
C1—C10—C11	120.96 (18)	C13—O3—Cu1^ii^	132.41 (14)

Symmetry codes: (i) *x*, −*y*+1/2, *z*+1/2; (ii) *x*, −*y*+1/2, *z*−1/2.
